# A Staged Biventricular Approach Combining the Starnes and Cone Procedures in Ebstein’s Anomaly: A Case Report and Literature Review

**DOI:** 10.3390/children12060782

**Published:** 2025-06-16

**Authors:** Paul Linnenbank, Daniel Biermann, Eike Philipp Schneider, Ida Hüners, Nora Lang, Fridrike Stute, Thomas S. Mir, Michael Hübler, Rainer Kozlik-Feldmann, Jakob Olfe

**Affiliations:** 1 Department of Pediatric Cardiology, Children’s Heart Clinic, University Heart & Vascular Center Hamburg, 20251 Hamburg, Germany; 2 Department of Congenital and Pediatric Heart Surgery, Children’s Heart Clinic, University Heart & Vascular Center Hamburg, 20251 Hamburg, Germany

**Keywords:** Ebstein’s anomaly, Starnes, cone reconstruction

## Abstract

**Background:** Severe neonatal Ebstein’s anomaly (EA) is associated with a high risk of mortality. A new therapeutic approach aims to combine the advantages of Starnes’ procedure in stabilizing critically ill neonates with the long-term superiority of biventricular physiology after cone reconstruction. **Case report**: The echocardiography of a male preterm (36 weeks’ gestation; birth weight 2400 g) demonstrated EA Carpentier type C, membranous pulmonary atresia, and hypoplastic pulmonary arteries (PAs). After undergoing the Starnes procedure postnatally, multiple dilatations of the AP shunt and the Starnes fenestration followed. Cone reconstruction was performed at 15 months of age. Surgical revision addressed tricuspid and pulmonary valve insufficiency and PA bifurcation stenosis. Subsequently, PA branch stenosis with severe impairment of right ventricular function and dilatation required stent implantation. At the last follow-up, at 3 years of age, the patient was asymptomatic with sufficient exercise tolerance. **Discussion**: The American Association for Thoracic Surgery recently recommended evaluating all Starnes patients for potential conversion to cone. Consequently, the Starnes procedure should be modified to facilitate subsequent biventricular correction. Both the optimal timing of conversion and the appropriate assessment to reliably evaluate feasibility and the prospects for success require further investigation. **Conclusions**: Conversion from Starnes to cone is technically feasible, even in cases of severe EA, prematurity, low birth weight, and additional cardiac comorbidities, and provides promising initial results. Further research is needed to define candidacy and the optimal timing of conversion, and to assess long-term outcomes. The high therapeutic effort and complexity make this treatment approach suitable only for quaternary centers.

## 1. Introduction

Ebstein’s anomaly [1] (EA) is a rare congenital malformation primarily affecting the tricuspid valve (TV) and the right ventricle (RV), characterized by marked morphological heterogeneity and variable clinical presentation. EA accounts for approximately 0.3–0.5% of congenital heart defects and about 40% of congenital TV malformations [2]. The hallmark feature is the apical and rotated displacement of the TV leaflets—most notably the septal and the inferior leaflet—from the valve plane into the RV, resulting from failed delamination during embryogenesis. This displacement leads to tricuspid regurgitation due to inadequate leaflet coaptation and the partial “atrialization” of the RV, characterized by dilation, thinning, and a loss of trabeculation [3].

Associated cardiac malformations include interatrial connections (over 90% of cases; atrial septal defect (ASD) or patent foramen ovale), often causing right-to-left shunting and cyanosis. Additional findings may include pulmonary stenosis or atresia, supraventricular arrhythmias, typically atrioventricular re-entry tachycardia (AVRT) via Wolff–Parkinson–White (WPW) accessory pathways, and lung hypoplasia secondary to severe right ventricular dilatation/cardiomegaly. Clinical presentation varies widely, from neonatal heart failure and demise to incidental diagnosis in adulthood [2].

Since its introduction, the Starnes procedure [4] has helped improve survival rates, particularly among severely affected neonates [5,6]. Modified over time, it encompasses the exclusion of the RV (eRV) at the anatomical level of the TV annulus using a fenestrated patch, the opening of the atrial septum, and the establishment of a source of pulmonary blood flow, typically through a systemic-to-pulmonary modified Blalock–Taussig–Thomas (mBTT) or central shunt [7]. Fenestration plays a critical role, especially in pulmonary atresia, as it prevents septal displacement by the expanding eRV at the expense of the left ventricle (LV) with the compression of the latter [3]. The Starnes procedure is particularly valued for its comparative simplicity, applicability to a wide range of valve morphologies, and independence from functional RV capacity [8]. However, at least until recently, the decision for Starnes’ procedure was considered a final commitment to univentricular circulation with the associated early and late complications [9,10].

Cone reconstruction [11] enables the preservation of biventricular physiology in EA. This approach involves the creation of a functional, cone-shaped valve using the dysplastic native tricuspid leaflets. Surgery includes meticulous mobilization of the leaflets from the functional annulus and detachment from their aberrant ventricular adhesions. Two vertical sutures are used to conjoin the septal leaflet medially and laterally to the anterior and inferior leaflet. The resulting eponymous cone is then reattached at the anatomical annulus. The atrial septal defect is closed in a flap-like fashion, allowing right-to-left shunting in the event of postoperative RV failure [12]. Despite its benefits [13,14,15,16], cone reconstruction is technically demanding with a substantial learning curve, which should be taken into account given the rarity of this malformation and is also reflected in the reported outcomes [8,14]. The individual morphology of some EA patients (e.g., the absence of a sufficiently large RV and patent right ventricular outflow tract (RVOT)) may also preclude this therapeutic approach, at least as primary care [8]. The same applies to very small and/or hemodynamically compromised newborns with still increased pulmonary vascular resistance [7]. In addition, it may be difficult to transfer such patients to centers of maximum medical care specializing in cone reconstruction in the first place [8].

In light of the above, da Silva et al. [17] recently proposed a hybrid strategy—delayed cone reconstruction following initial stabilization with the Starnes procedure. A promising concept, as this two-stage approach may isolate the early benefits of Starnes in critically ill neonates with EA from the long-term disadvantages of permanent Fontan circulation and subsequently combine them with the benefits of 1.5 or biventricular physiology created by cone repair. Notably, it has been reported that the RV undergoes favorable remodeling after Starnes and thus may successively be enabled to generate higher pressure gradients over time [8,17]. Furthermore, patients are also given more time for leaflet tissue development, lung maturation, and the growth of the pulmonary arteries, making them possible candidates for RV recruitment by cone repair, for which they were not yet eligible postnatally [8,18]. While early outcomes are encouraging [7,17,18,19], long-term longitudinal studies do not exist to date. It is still unclear what preconditions patients need to meet to benefit (particularly) from this two-stage treatment approach and which time window should be complied with for the recruitment of the RV after Starnes [7,17,18].

The following report illustrates our institutional experience with this treatment approach based on the case of a male child with EA Carpentier type C.

## 2. Case Report

**Figure 1:**
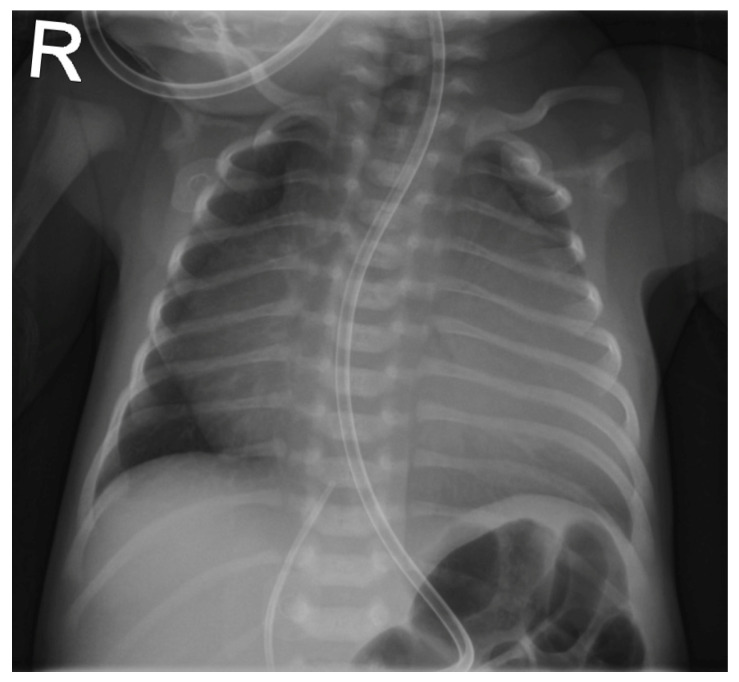
Chest radiograph showing severe cardiomegaly; immediately postpartum.

**Figure 2:**
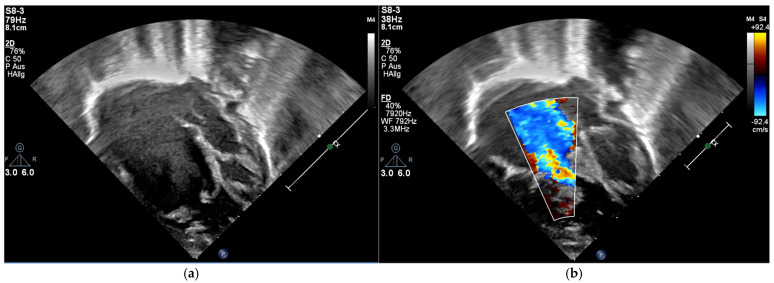
Echocardiography, four-chamber view; (**a**) the apical displacement of the tricuspid valve; (**b**) color-flow Doppler showing severe tricuspid regurgitation; immediately postpartum.

The patient was born late-preterm by cesarean section (36 + 3 weeks’ gestation, birth weight 2400 g, APGAR 6/9/9, umbilical cord pH = 7.38) with prenatally diagnosed EA; we started immediate, uncomplicated primary care with CPAP respiratory support, umbilical vein catheter and prostaglandin E (PGE) infusion for ductus-dependent lung perfusion. With stable hemodynamics, the neonate was transferred to our pediatric cardiac intensive care unit (pCICU). At admission, the patient exhibited independent feeding ability, a heart rate of 150 bpm, pre- and postductal oxygen saturation was >85%, and additional oxygen was required until the third day of life. ECG showed sinus rhythm with P-dextroatriale, QRS of approx. 80 ms, and no arrhythmia. On the second day postpartum, Troponin I and proBNP were measured as 9 pg/mL and 27,065 ng/L, respectively. Mild signs of heart failure were treated with diuretics. Echocardiography confirmed severe EA, consistent with Carpentier type C, with RA/(RV + LA + LV) > 1 (RA = right atrium; LA = left atrium), ASD type II of 10 mm, and the prolapse of the anterior leaflet of the TV into the RVOT leading to severe insufficiency with a coaptation defect and a V. contracta of 1.2 cm. Additionally, membranous pulmonary atresia with preserved confluence was found. The pulmonary arteries (PAs) were hypoplastic, with left PA (LPA) = 2.7 mm (*z* = −2.3) and right PA (RPA) = 3.2 mm (*z* = −1.7). The left-sided heart segments did not show any malformation, insufficiency, or stenosis, and their function was deemed to be satisfactory (fractional shortening (FS) = 22.6%; see Figure 1 and Figure 2, Supplementary Materials, Figure S1).

The pronounced EA findings, the child’s prematurity, and low birth weight, as well as the combination of membranous pulmonary atresia and small-caliber PAs, precluded cone reconstruction. Therefore, on the tenth day of life, Starnes palliation [4] was performed. The procedure included fenestrated TV closure (Gore-Tex^®^ patch, 3.5 mm/perforation 2.8 mm, W.L. Gore & Associates Inc., Newark, DE, USA) preserving the dysplastic native leaflets, atrioseptectomy, central aortopulmonary (AP) shunt placement (aorta on main pulmonary artery (MPA) trunk, side-to-side; Gore-Tex^®^, 3.5 mm, W.L. Gore & Associates Inc., Newark, DE, USA; clipped), and the excision of the free wall of the RA. The patient was transferred to the pCICU with an open thorax. Confronted with a tachycardic junctional replacement rhythm, temperature optimization, continuous drip infusion of amiodarone, and AAI overpacing were performed, which enabled rate control. Chest closure and extubation were carried out on postoperative days (PODs) 3 and 7, respectively. Oxygen dependency persisted beyond transfer to the regular care unit, with mean transcutaneous saturation levels of 75–80%. The respiratory pattern showed mild tachypnea, the postoperative regression of cardiomegaly, and mild retrocardiac dystelectasis. By the time of discharge to ambulatory care in the middle of the second month of life, the child’s condition had gradually stabilized, and signs of heart failure had steadily decreased (Ross score 2/12). Permanent platelet aggregation inhibition with ASA was initiated. Pronounced supraventricular extrasystolia (SVE) with short atrial runs required intensified bisoprolol therapy, under which the findings improved; Holter ECG at discharge showed only occasional SVE.

Echocardiography showed a decompressed RV with bidirectional shunt flow across the fenestrated Starnes patch and positive septal shift (RV pressure = 7 mmHg). The LV showed preserved function (FS = 36%, mitral annular plane systolic excursion (MAPSE) = 6 mm) without valvular insufficiency. The shunt perfused both PAs adequately, although flow was preferentially directed to the LPA. The RPA remained narrow (3 mm).

**Figure 3:**
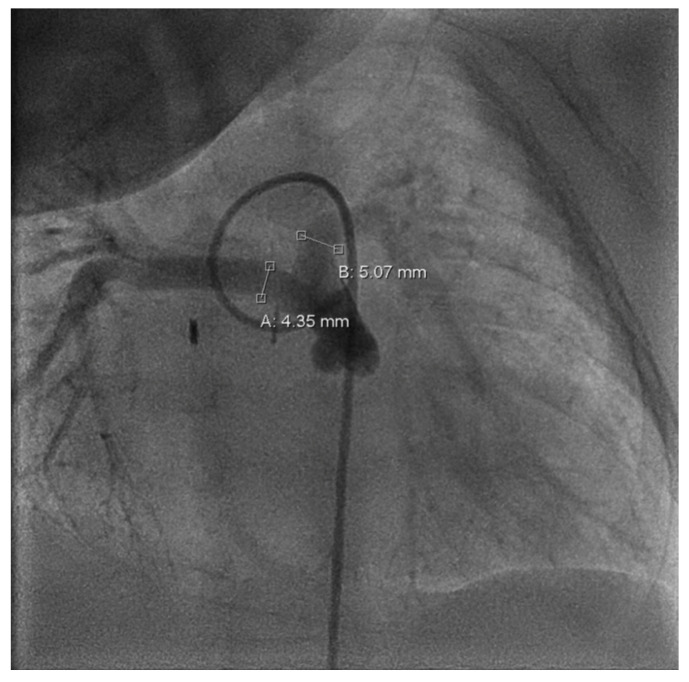
Cardiac catheterization, a.p.; measurement of the left pulmonary artery (=B: 5.07 mm) and right pulmonary artery (=A: 4.35 mm) diameter.

Follow-up in the third month of life confirmed preserved LV function (FS = 38%) and bidirectional shunt flow across the Starnes patch with an estimated RV pressure of 10 mmHg and a well-perfused AP shunt with a stable gradient of 4 m/s. Both hypoplastic PAs were perfused (LPA > RPA; LPA = 4.9 mm, RPA = 2.3 mm). Due to increasing LPA/RPA imbalance and filiform, laminar RPA flow, sectional imaging was performed via magnetic resonance imaging (MRI). Because of the bilaterally small-caliber PAs without dilatable local stenosis and insufficient RV volume for biventricular correction, we opted for interventional dilation of the AP shunt clip. As part of the corresponding cardiac catheterization (CC) at the beginning of the fourth month of life, several balloon dilatations of the AP shunt in clip position to a maximum of 4 mm were performed. The PAs appeared to be more prominent on CC than depicted in MRI (LPA = 5 mm, *z =* −0.2; RPA = 4.3 mm, *z =* −1.1; see Figure 3).

To prevent increasing stasis in the RV and to provide an additional growth stimulus to buy time before planned bi- or 1.5-ventricular reconstruction, the Starnes fenestration was dilated with two cutting balloons at the beginning of the sixth month of life (see Supplementary Materials, Figure S2).

At the beginning of the tenth month of life, decreasing oxygen saturation (S_p_O_2_ = 71–81%) prompted another CC. A 4 mm balloon was used for twofold dilation of the AP shunt. The aortic anastomosis was found to have an outlet stenosis, which could only be dilated to a limited extent. We also carried out hemodynamic measurements and angiography. The RV was encouragingly well developed, with an intraventricular pressure of 32/0/8 mmHg. The PAs still appeared small but also better developed (LPA = 5.3 mm, *z* = −0.4; RPA at the outlet = 4.5 to 4.7 mm, *z* = −1.9 to −1.8, widening to distal 6.3 mm, *z* = 0; MPA narrow with 6.2 to 6.8, *z* = −3.4 to −2.8), PA mean pressure = 20 mmHg, central venous pressure (CVP) = 9–10 mmHg. On echocardiography, the RV was visually and numerically restricted as far as its function could be assessed, with tricuspid annular plane systolic excursion (TAPSE) = 6 mm and ejection fraction (EF) = 30%, RV end-diastolic volume (RVEDV) = approx. 15–17 mL, RV end-diastolic diameter (RVEDD) = 13 mm. The Starnes membrane appeared at TV level with pendulum flow across the fenestration, measured with an RV systolic pressure (RVSP) of 15 mmHg + CVP. The native TV showed a septal leading jet of insufficiency with a tightly adherent leaflet; the anterior leaflet appeared large/prolapsed, the posterior leaflet rather small and without significant apical displacement.

By the end of the eleventh month of life, oxygen saturation had fallen to pre-interventional levels (S_p_O_2_ = 75–82%), prompting the need for a fundamental decision regarding further treatment. RV function appeared improved with a possible pressure build-up of about 30 mmHg; MRI volumetry showed an RVEDV of 22.1 mL (RVEDV/body surface area (BSA) = 64.0 mL/m^2^); LV end-diastolic volume (LVEDV) was measured with 33.4 mL (LVEDV/BSA = 97.0 mL/m^2^). In conjunction with RV size and PA diameter, a biventricular correction in the form of a cone reconstruction was considered feasible and worth striving for (see Supplementary Materials, Figure S3).

At the end of the 15th month of life, with a body weight of 7.3 kg, the operation, including resection of the fenestrated Starnes patch at the TV, a cone-shaped reconstruction of the TV according to da Silva [11], the atrial septal closure of the common atrium via a patch (Gore-Tex^®^, W.L. Gore & Associates Inc., Newark, DE, USA), the placement of a valved right ventricle-to-pulmonary artery (RV-PA) conduit (Contegra^®^, 12 mm, Medtronic Inc., Minneapolis, MN, USA), and the takedown of the AP shunt, was performed. Following postoperative ventilation, extubation was successful on the first POD. Hemodynamic stabilization was achieved with high catecholaminergic support, but otherwise without complications. Echocardiography showed mild congestion of the hepatic veins; the TV was competent after cone repair with monophasic inflow and a mean gradient of 7 mmHg. RVSP was not derivable. Overall, the RV was still significantly restricted in its function. The conduit valve appeared without higher-grade insufficiency or stenosis.

At the age of one year and six months, acute right heart decompensation with increasing dilatation and functional impairment of the RV occurred. Echocardiography showed moderate to severe TV insufficiency with RVSP = 22 mmHg, dilation of the RV-PA conduit, pulmonary valve (PV) insufficiency due to impaired pocket coaptation, and a severe bifurcation stenosis of about 3–4 mm on both sides with flow acceleration to 2.6 m/s. LV function was preserved, with low-grade mitral valve insufficiency. There was no pleural or pericardial effusion, but distinct ascites. CC showed a low cardiac index of 2.47 l/min/m^2^, as well as significantly increased filling pressures of 30 mmHg in the RV, in which there was 2/3 systemic pressure with 60 mmHg vs. 82 mmHg systolic pressure in the descending aorta. Across the conduit, there was an antegrade gradient of 40 mmHg and low mean PA pressures of 17 mmHg further distally due to the severe stenosis of the peripheral PAs in the area of the distal anastomosis. Interventional treatment via CC was considered unfeasible, making surgery inevitable. Subsequently, TV reconstruction via cleft closure, bifurcation plasty of the peripheral Pas, and the replacement of the valved RV-PA conduit (Contegra^®^, 12 mm, Medtronic Inc., Minneapolis, MN, USA) were performed. Postoperatively, the child was transferred intubated and ventilated and again required high-dose catecholaminergic circulatory support. Extubation to non-invasive ventilation was performed in the fifth postoperative hour (see Supplementary Materials, Figure S4).

**Figure 4:**
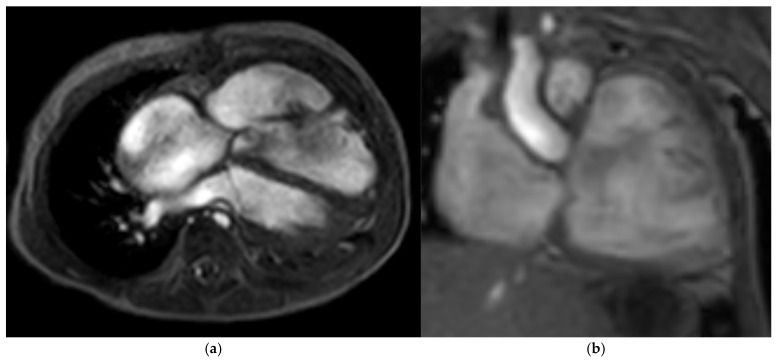
MRI of the right heart and the tricuspid valve; the right atrium and right ventricle present with distinct dilatation; (**a**) axial plane, (**b**) coronal plane; 14 months after cone reconstruction, age 2 and a half years.

At follow-up at nearly two years of age (five months after the revision), a slight acceleration of flow (mean 4 mmHg) across the TV accompanying a moderate TV insufficiency with RVSP of 35 mmHg + CVP was still demonstrable on echocardiography. The mitral valve showed a regular, biphasic inflow with grade I insufficiency. The RV continued to exhibit marked functional impairment due to global mural dysfunction and dilation with ventricular wall thinning (TAPSE = 5 mm, RVEDD = 35 mmHg). The RA presented with the known significant dilation. The conduit valve appeared to function adequately showing mild insufficiency and no stenosis. The outlets of the LPA and RPA were measured as 1.3 m/s and 1.1 m/s, respectively (see Figure 4).

**Figure 5:**
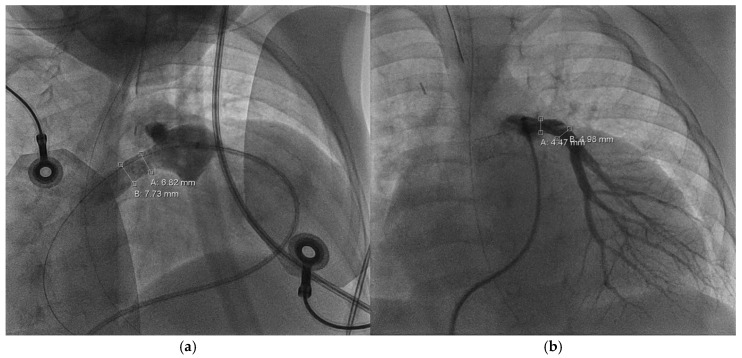
Cardiac catheterization, a.p.; (**a**) the stenting of the right pulmonary artery; (**b**) the left pulmonary artery for comparison; 18 months after cone reconstruction.

**Figure 6:**
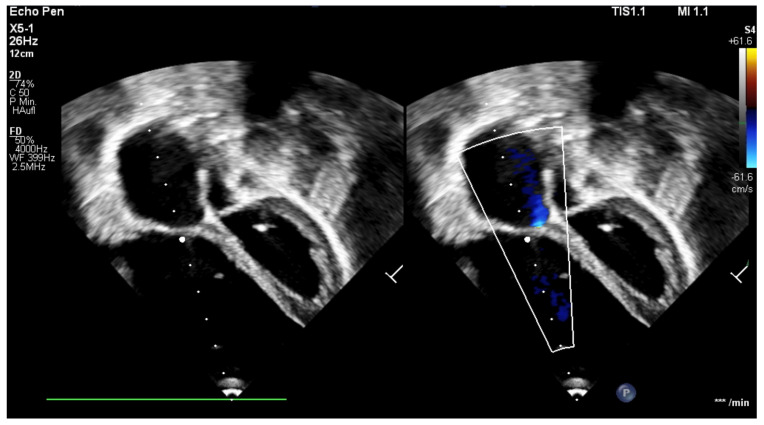
Echocardiography, four-chamber view; 18 months after cone reconstruction, 1 week after cardiac catheterization with stent implantation in the right pulmonary artery. *** = heart beats/minute, heart beats were not derived in this echocardiography.

Entering the ninth month of the third year of life, CC revealed severe RPA branch stenosis (3.5 mm, *z* = −5.1), along with a dilated RV (EDV = 219 mL/m^2^) of severely impaired function (EF = 11%), requiring the implantation of a 6 × 12 Formula^®^ stent (Cook Medical Inc., Bloomington, IN, USA). LPA measured 4.9 mm (*z* = −2.2) without branch or peripheral stenosis. The cardiac index was 4.6 l/min/m^2^ and CVP dropped from 18 to 5 mmHg after stent implantation. Within weeks after the procedure, RV function and dilatation improved significantly (see Figure 5 and Figure 6).

At the last follow-up at three years of age, the patient was in good general condition (RR = 102/58, S_p_O_2_ = 98%, respiratory rate = 23/min, body weight = 10,8 kg) with no obvious signs of reduced exercise tolerance. Further development remains to be seen. (See Supplementary Materials, Figure S5, for a step-by-step summary table.)

## 3. Discussion

Neonates with symptomatic EA represent an exceedingly complex and heterogeneous patient population, posing exceptional challenges in clinical management. Nearly half (46%) of newborns diagnosed with EA require some form of neonatal intervention [5], for which LaSala et al. [20] reported a mortality rate of 37.5% within a 1-year follow-up. This underscores the critical nature of early decision-making, as the narrow therapeutic window at this stage leaves little room for a trial-and-error approach. At the same time, however, there are still no universally accepted treatment guidelines, not least due to the rarity of the condition and the absence of randomized controlled clinical/surgical trials. As a result, therapeutic approaches vary considerably depending on location and appear to be based primarily on the physicians’ individual experience and personal preference [8]. In an effort to provide greater clarity, the American Association for Thoracic Surgery (AATS) recently published a consensus document summarizing the current evidence base alongside expert experience and opinion regarding the management of neonates with symptomatic EA [21].

The Starnes procedure [4] has proven its value in treatment and survival, particularly in critically ill, hemodynamically unstable neonates with EA [5,6]. The AATS considers Starnes’ therapeutic approach superior to others in patients with a neonatal circular shunt and/or refractory cardiogenic shock (e.g., those on inotropic support, ongoing PGE infusion due to ductus-dependent pulmonary perfusion, and positive pressure ventilation). It seems to provide the most reliable initial stabilization, respectively, palliation with an expected mortality rate of 10–20% [21]. In a single-center study by Kumar et al. [6], 22 of 27 patients survived to hospital discharge following Starnes palliation, including one who underwent non-fenestrated RV exclusion. Of those survivors, 21 progressed to a Glenn procedure and 20 to Fontan completion. At a median follow-up of 7 years, all patients exhibited normal LV function; 95% showed NHYA class I symptoms, 90% were in sinus rhythm (two patients required pacemakers), and none were dependent on long-term antiarrhythmics [6]. A more recent study by Freud et al. [5] even reported a 0% in-hospital mortality rate in a multicenter cohort of 15 patients who underwent the Starnes procedure [5]. Cleveland and Starnes himself [22], as well as our case, demonstrate the applicability and utility of the procedure even in cases of prematurity and extremely low birth weight; populations in whom alternative options such as ductal stenting or isolated mBTT shunt [21] are either contraindicated or pose elevated risk. However, the aging population of Fontan recipients demonstrates the severe limitations of univentricular circulation. A Danish 30-year follow-up study by Kelly et al. [10] found a 79% freedom from death, transplant, or Fontan takedown, and significant morbidity—including ventricular dysfunction, arrhythmia, significant atrioventricular valve regurgitation, and protein-losing enteropathy—affecting 38% of survivors, alongside a progressive decline in exercise capacity with age [10]. In contrast, cone reconstruction has yielded excellent long-term results, with low-to-absent mortality [14,16,23,24] rates and minimal rates of reinterventions [13,16,24] or more than mild-to-moderate tricuspid regurgitation [13,14,16]. The functional status is also reported to be preserved or good in the vast majority of patients [16], making this an attractive definitive repair strategy. Nevertheless, it is important to note that most patients included in these studies were not considered high-risk. Thus, extrapolating these outcomes to symptomatic neonates—such as the case presented here—requires caution. The AATS advises that, despite few reports of successful cone reconstructions in high-risk patients, the biventricular approach according to da Silva [11] should generally be pursued secondarily after initial Starnes palliation [21]. As described above, the intricacies of cone repair—including a considerable learning curve [8,14]—often conflict with the clinical urgency encountered in neonatal EA, particularly in institutions without specialized surgical experience. In some instances, the EA’s individual morphology or the RV’s functional capacity preclude primary biventricular repair altogether. Mandatory delivery in or transfer to a quaternary center is also not always possible for medical or logistical reasons, especially in areas with limited access to health care.

Stated simply: for neonates with symptomatic EA in whom cone repair is contraindicated or deemed unfeasible, Starnes’ procedure is generally the preferred initial intervention. Uncertainty arises in “borderline” cases, such as neonates with challenging anatomy but initially stable hemodynamics. These patients will become unstable at some point—the question is how best to care for them in the meantime. Ultimately, the treatment decision (at least to date) remains a matter of weighing up the options and can only be made on a multidisciplinary and individual basis. In the future, a two-stage approach—beginning with Starnes surgery to achieve stabilization, followed by delayed bi- or 1.5-ventricular repair—may not turn out to be the only right one, but will rarely be wrong.

What implications arise for the management of EA from Starnes and beyond? The AATS [21] recommends all patients who have undergone and will undergo Starnes surgery to be evaluated for possible conversion to cone reconstruction. Consequently, the Starnes procedure should be modified to facilitate subsequent biventricular correction. This includes the optimal possible preservation of the TV and PV, as well as ensuring age-appropriate growth of the eRV [21]. Therefore Dobson et al. [7] advocate the use of a Starnes patch made of polytetrafluoroethylene (PTFE) to minimize adhesions to the TV. For better preservation, patch and fenestration placement should avoid TV leaflets and the patch should be sutured slightly above the TV to facilitate eventual takedown and to reduce the risk of causing AV block [7]. Furthermore, the AATS [21] proposes positioning the coronary sinus orifice below the patch, as leaving it on the eRV side may foster RV development, while PA patch closure should be performed above the valve to preserve its integrity. Close clinical and imaging monitoring, including transthoracic echocardiography and MRI as part of standard protocol, is recommended afterward and later in the course of treatment [21].

After the fenestrated closure of the TV, there is a risk of progressive RV shrinkage. When choosing the right timing for conversion to cone, it should be ensured that it is performed before any irreversible remodeling of the eRV occurs [21]. In our case, balloon dilatation of the Starnes fenestration increased RV perfusion without overloading, thereby promoting RV growth. In patients with more favorable PA anatomy, this may also be a way to delay conversion until pulmonary resistance declines sufficiently to open up the option of Glenn/1.5-ventricle circulation, should this become necessary. The AATS currently identifies infancy (<1 year) or shortly thereafter as a potentially favorable window for conversion [21], yet further research is needed to establish evidence-based timing.

Prior to possible conversion, Dobson and colleagues [7] focused especially on the following imaging factors: “(1) tethering of anterior leaflet to RV free wall, (2) angle of TV rotation, (3) septal leaflet morphology, (4) displacement/attachment of the inferior leaflet, (5) number of TV orifices and (6) type of distal valve attachment (focal, hyphenated, or linear)” [7].

Comprehensive echocardiographic evaluation should incorporate findings from all prior imaging, including pre-Starnes or even fetal echo, as this may allow for a more accurate assessment of TV morphology and RV size and function, as compared to the underfilled eRV [19]. While pre-cone RVSP estimates via regurgitation across the Starnes fenestration may offer insights into RV contractile potential, there seems to be little or no correlation with the success of conversion [7,18]. Likewise, in our case, the actual significance and reliability of such measurements, for instance, the MRI volumetry of the eRV we performed, was subject to discussion within our interdisciplinary team. Cardiac catheterization may offer the most accurate assessment of the actual size of the eRV [19]. Such a preoperative assessment can be helpful in evaluating the feasibility of the procedure. Nevertheless, precise criteria, including cut-off values, for determining conversion candidacy, and thus specific indications and contraindications, remain undefined and need to be investigated further.

According to Dobson et al. [7], impediments to successful conversion include RV dysfunction, previous repair attempts, abnormal PV morphology, and pronounced Ebstein findings. However, as we were able to show in our case with Carpentier type C, membranous pulmonary atresia, and hypoplastic pulmonary arteries, the limits of possible conversion, determined by the EA’s anatomical conditions, appear to be, if not infinite, at least very wide. Instead, extracardiac factors such as extreme prematurity, low birth weight, genetic syndromes, or severe comorbidities may more often represent true contraindications.

Da Fonseca da Silva and her Pittsburgh colleagues recently reported promising results from 17 consecutive conversions. All patients were discharged in good clinical condition, with 6 exhibiting trivial TV insufficiency, 9 presenting with mild, and 2 with mild-to-moderate regurgitation [19]. While encouraging, these findings must be balanced against the high cumulative treatment burden associated with the staged conversion pathway. Surgical complexity, the need for multiple (catheter) interventions, and the potential for reoperation may all significantly impact morbidity and quality of life. Thus, from our point of view, conversion should only proceed after thorough, multidisciplinary deliberation and informed consent. Families must be counseled not only on potential benefits but also on the inherent risks and uncertainties of this evolving therapeutic approach.

## 4. Conclusions

Conversion from Starnes to cone represents an emerging therapeutic paradigm that may combine the advantages of Starnes [4] in stabilizing critically ill neonates with the long-term superiority of biventricular physiology achieved through cone reconstruction [11]. With sufficient experience, it is technically feasible even in neonates with pronounced EA findings harboring complex anatomical conditions, prematurity, and low birth weight, and yields encouraging early results. Precise indications and the optimal timing of conversion require further investigation. Long-term outcomes are yet to be characterized. High procedural complexity with substantial risks and intensive resource requirements should be considered, making this treatment approach suitable only for centers of maximum medical care.

## Data Availability

The original contributions presented in this study are included in the article. Further inquiries can be directed to the corresponding author.

## Supplementary Materials

The following supporting information can be downloaded at: https://www.mdpi.com/article/10.3390/children12060782/s1, Figure S1: Echocardiography, a short-axis view showing the dilated right atrium and the atrialized right ventricle, immediately postpartum; Figure S2: Echocardiography, four-chamber view, 1 day after the dilatation of the Starnes fenestration; Figure S3: Chest radiograph, 12 months after Starnes operation; Figure S4: Echocardiography, four-chamber view, 6 months after cone reconstruction; Figure S5: Step-by-step summary of the major interventions and the ages at which they were performed.

## Abbreviations

The following abbreviations are used in this manuscript:

AAI	single-chamber pacing
AATS	American Association for Thoracic Surgery
ACE	angiotensin-converting enzyme
AP	Aortopulmonary
ASA	acetylsalicylic acid
ASD	atrial septal defect
AVRT	atrioventricular re-entry tachycardia
BSA	body surface area
CC	cardiac catheterization
CPAP	continuous positive airway pressure
CVP	central venous pressure
EA	Ebstein’s anomaly
ECG	electrocardiogram
EF	ejection fraction
eRV	excluded right ventricle
FS	fractional shortening
LA	left atrium
LPA	left pulmonary artery
LV	left ventricle
LVEDV	left ventricular end-diastolic volume
MAPSE	mitral annular plane systolic excursion
mBTT	modified Blalock–Taussig–Thomas
MPA	main pulmonary artery
MRI	magnetic resonance imaging
PA	pulmonary artery
pCICU	pediatric cardiac intensive care unit
PGE	prostaglandin E
POD	postoperative day
PTFE	Polytetrafluoroethylene
PV	pulmonary valve
RA	right atrium
RPA	right pulmonary artery
RV	right ventricle
RVEDD	right ventricular end-diastolic diameter
RVEDV	right ventricular end-diastolic volume
RVOT	right ventricular outflow tract
RV-PA	right ventricle to pulmonary artery
SVE	supraventricular extrasystolia
TAPSE	tricuspid annular plane systolic excursion
TV	tricuspid valve
WPW	Wolff–Parkinson–White
