# Supplementary material for: A Staged Biventricular Approach Combining the Starnes and Cone Procedures in Ebstein’s Anomaly: A Case Report and Literature Review
Source: Children (Basel). 2025 Jun 16;12(6):782. doi: 10.3390/children12060782 (PMC12191472; doi:10.3390/children12060782)
Supplement: Supplementary file 1 [file children-12-00782-s001.zip › children-3671303-supplementary.pdf]

Supplementary Materials:

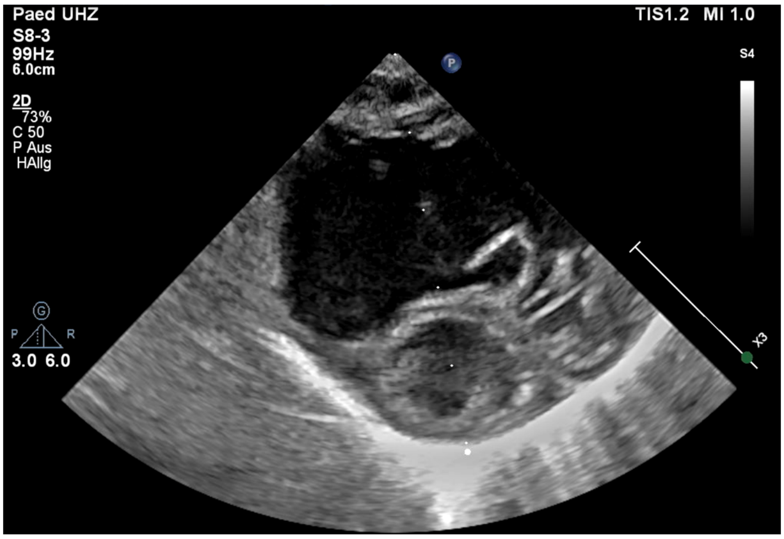

Figure S1. Echocardiography, short axis view showing the dilated right atrium and the atrialized right ventricle, immediately postpartum.

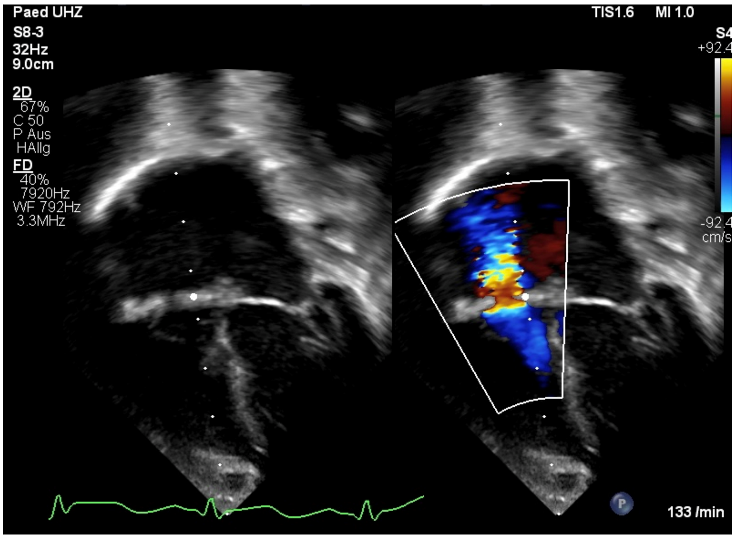

Figure S2. Echocardiography, four chamber view, 1 day after dilatation of the Starnes fenestration.

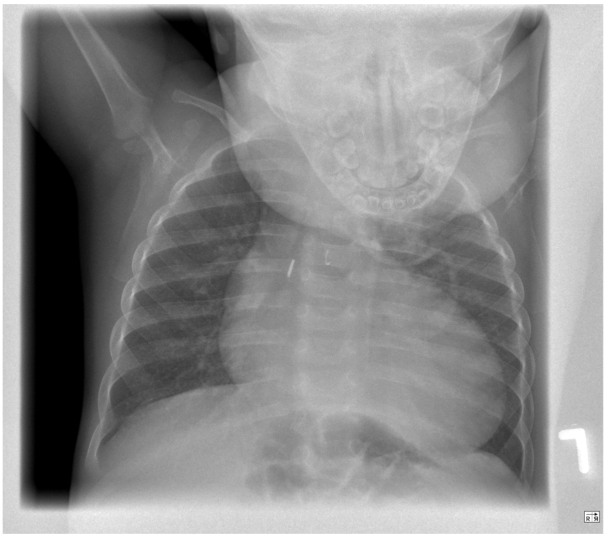

Figure S3. Chest radiograph, 12 months after Starnes operation.

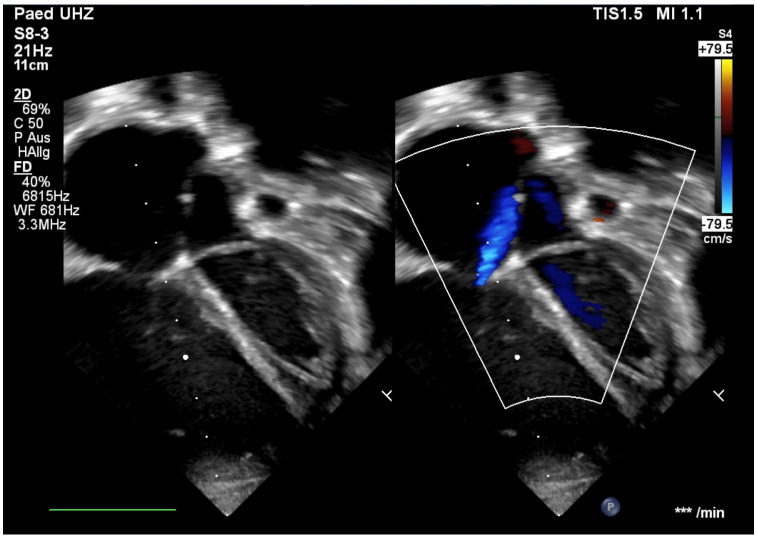

Figure S4. Echocardiography, four chamber view, 6 months after Cone reconstruction.

Age	Intervention
**Day 10**	Starnes procedure with fenestrated TV patch and 3.5 mm AP shunt
**4th month**	First balloon dilatation of AP shunt (up to 4 mm)
**6th month**	Cutting balloon dilatation of Starnes fenestration
**10th month**	Second AP shunt dilatation for outlet stenosis
**15th month**	Cone reconstruction of TV, RV-PA conduit (12 mm), AP shunt removal
**18th month**	Revision: TV repair, PA bifurcation plasty, RV-PA conduit replacement
**33rd month**	RPA stent implantation (6×12 mm)
**36th month**	Stable clinical status with restrictive RV function

Figure S5. Step-by-step summary of the major interventions and the ages, at which they were performed.
